# Intra‐strain biological and epidemiological characterization of plum pox virus

**DOI:** 10.1111/mpp.12908

**Published:** 2020-01-24

**Authors:** Kensaku Maejima, Masayoshi Hashimoto, Yuka Hagiwara‐Komoda, Akio Miyazaki, Masanobu Nishikawa, Ryosuke Tokuda, Kohei Kumita, Noriko Maruyama, Shigetou Namba, Yasuyuki Yamaji

**Affiliations:** ^1^ Department of Agricultural and Environmental Biology Graduate School of Agricultural and Life Sciences The University of Tokyo Tokyo Japan; ^2^ Department of Sustainable Agriculture College of Agriculture, Food and Environment Sciences Rakuno Gakuen University Ebetsu Hokkaido Japan

**Keywords:** evolutionary epidemiology, intra‐strain variation, plum pox virus, positive selection, reversible evolution, transient attenuation

## Abstract

Plum pox virus (PPV) is one of the most important plant viruses causing serious economic losses. Thus far, strain typing based on the definition of 10 monophyletic strains with partially differentiable biological properties has been the sole approach used for epidemiological characterization of PPV. However, elucidating the genetic determinants underlying intra‐strain biological variation among populations or isolates remains a relevant but unexamined aspect of the epidemiology of the virus. In this study, based on complete nucleotide sequence information of 210 Japanese and 47 non‐Japanese isolates of the PPV‐Dideron (D) strain, we identified five positively selected sites in the PPV‐D genome. Among them, molecular studies showed that amino acid substitutions at position 2,635 in viral replicase correlate with viral titre and competitiveness at the systemic level, suggesting that amino acid position 2,635 is involved in aphid transmission efficiency and symptom severity. Estimation of ancestral genome sequences indicated that substitutions at amino acid position 2,635 were reversible and peculiar to one of two genetically distinct PPV‐D populations in Japan. The reversible amino acid evolution probably contributes to the dissemination of the virus population. This study provides the first genomic insight into the evolutionary epidemiology of PPV based on intra‐strain biological variation ascribed to positive selection.

## Graphical abstract

This is the first evidence of intra‐strain variation in plum pox virus ascribed to positive selection, especially reversible evolution that contributes to overcoming bottleneck by plant quarantine.

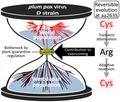

## 1 INTRODUCTION

The emergence of unexpected viral diseases often results in considerable yield losses in crops (Picó *et al.*, 1996; Gonsalves, 1998; Cambra *et al.*, 2006; Legg *et al.*, 2006; Moreno *et al.*, 2008). For the control of such threats, it is important to understand the geographical distribution and biological features of emerging viruses through epidemiological approaches. In particular, large‐scale epidemiological surveys analysing genomic signatures of the virus isolates have been the most promising approach for understanding viral strains, distribution pathways, and related ecological and evolutionary processes (Traoré *et al.*, 2009; Picard *et al.*, 2017).

Plum pox virus (PPV), an aphid‐borne RNA virus belonging to the genus *Potyvirus*, is the causal agent of a serious viral disease of stone fruit trees (*Prunus* species), known as sharka (García *et al.*, 2014). PPV is one of the most important and well‐studied plant viruses because of its economic impact (Scholthof *et al.*, 2011). Despite stringent quarantine regulations, it had spread throughout a large part of Europe by the 1980s, and has been detected (with a restricted distribution) in South and North America, Africa, and Asia within the past three decades (Rimbaud *et al.*, 2015b). Through extensive research efforts to understand the virus diversity, at least 10 monophyletic strains of PPV (D, M, EA, C, Rec, W, T, CR, An, and CV) have been identified to date (García *et al.*, 2014; Chirkov *et al.*, 2018). Because some strains possess distinct biological properties, such as host preference, aphid transmissibility, disease symptomatology, and geographic distribution (James *et al.*, 2013; Rimbaud *et al.*, 2015b; Sihelská *et al*., 2017), strain typing has been the sole approach to the characterization of PPV isolates or populations of interest (EPPO, 2006; IPPC, 2018). Meanwhile, biological variation among isolates or populations in a single strain has been recognized as another important but unsolved aspect of PPV epidemiology (Candresse and Cambra, 2006; James *et al.*, 2013; García *et al.*, 2014; Rimbaud *et al.*, 2015b). Several studies have revealed intra‐strain variation in biological properties, such as host preference (Maiss *et al.*, 1995; Dallot *et al.*, 1998), aphid transmissibility (Deborre *et al*., 1995; Glasa *et al.*, 2004; Schneider *et al.*, 2011), and competitiveness (Glasa *et al.*, 2010), among PPV isolates. Although these intra‐strain biological variations are believed to be due to viral genetic diversity (James *et al.*, 2013), existing studies have not elucidated the correlations among them based on genome sequences of PPV isolates, with the exception of certain non‐aphid‐transmissible isolates with mutations in the Pro‐Thr‐Leu (PTK) and Asp‐Ala‐Gly (DAG) motifs, which are responsible for aphid transmission of potyvirus (Atreya *et al.*, 1991; Peng *et al.*, 1998) in the helper component proteinase (HC‐Pro) (Glasa *et al.*, 2004) and coat protein (CP) (Maiss *et al.*, 1995; Kamenova *et al.*, 2002), respectively. Therefore, it has been suggested that biological information for a range of isolates from each PPV strain is unlikely to be available in the immediate future (Candresse and Cambra, 2006).

PPV‐Dideron (D), the most widely distributed strain, is widespread throughout Europe and has also been detected in South and North America, Africa, and Asia (EPPO, 2006; Chirkov *et al.*, 2016; Oh *et al.*, 2017), including Japan (Maejima *et al.*, 2010; Maejima *et al.*, 2011). In this study, to investigate intra‐strain biological variation in PPV‐D, we performed molecular epidemiological analysis based mainly on virus samples collected in Japan. Based on complete nucleotide sequence information, we detected five codons under positive selection. One codon was suggested to be responsible for intra‐strain biological properties that could be involved in the evolutionary epidemiology of PPV.

## 2 RESULTS

### 2.1 Sequence properties of Japanese PPV isolates

**Figure 1:**
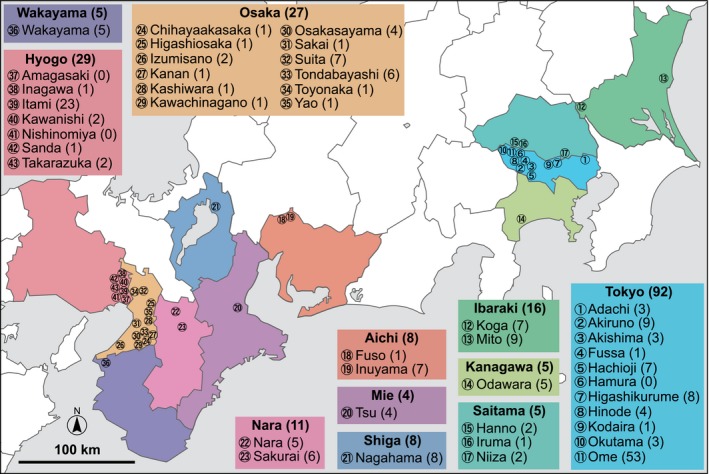
The distribution map of PPV in Japan shown based on the 2014 nationwide survey. In total, 210 PPV‐infected *Prunus* spp. were collected from 40 of 43 municipalities (plotted on the map) in 11 prefectures where PPV was detected between 2008 and 2014. They included 37 samples reported in our previous study (Maejima *et al*., 2011). Numbers in parentheses indicate the numbers of sequenced samples. Detailed information for each sample is shown in Table S1

In Japan, PPV‐D was first detected from Japanese apricot trees (*Prunus mume*) in Tokyo in the spring of 2009 (Maejima *et al.*, 2010). A preliminary survey identified PPV‐infected trees in several areas in and around Tokyo in the summer of 2009, and these PPV isolates were found to be monophyletic (Maejima *et al.*, 2011). Subsequently, annual nationwide surveys involving the inspection of 2.7 million stone fruit trees identified more than 29,000 PPV‐infected trees. Large PPV outbreaks were detected not only in eastern Japan (near Tokyo), but also in western Japan (near Osaka), several hundred kilometres from Tokyo (Figure 1) (MAFF, 2019).

To elucidate the diversity and phylogenetic relationships of PPV in Japan, we determined the genome sequences of 173 Japanese PPV isolates collected from most of the cities/towns where PPV was detected (Figure 1 and Table S1) between 2009 and 2014. All isolates shared high sequence identity (>98%) with the 37 Japanese PPV‐D isolates reported previously (Maejima *et al.*, 2011), indicating that they also belong to the PPV‐D strain. Most of the 173 viral genomes had the same length, with four exceptions: a single nucleotide deletion/insertion was observed in the 5′‐ or 3′‐untranslated region (UTR) of four isolates. Amino acid motifs associated with aphid transmission (Lys‐Ile‐Thr‐Cys [KITC] and PTK motifs in the HC‐Pro region, and the DAG motif in the CP region) (Ng and Falk, 2006) were conserved among all of the Japanese isolates, with the exception of the Wa7 isolate, which contained asparagine (N) instead of aspartic acid (D) in the conserved DAG motif. Interestingly, while our previous study showed that the five nucleotide positions (nt 657, 1,598, 2,928, 5,246, and 7,145 of the reference genome sequence of the Ou1 isolate [AB545926]) were conserved only among Japanese isolates (Maejima *et al.*, 2011), they were not necessarily conserved among the 173 newly sequenced Japanese isolates, casting doubt on our previous hypothesis that PPV‐D found in Japan originates from a single introduction of infected plant material.

### 2.2 Phylogenetic relationships among global populations of PPV‐D

To elucidate the origin and epidemiological dynamics of PPV‐D in Japan, we next conducted a phylogenetic analysis of all Japanese isolates along with non‐Japanese PPV‐D isolates. In total, 257 full genome sequences of the PPV‐D isolates (210 Japanese and 47 non‐Japanese) were used in this analysis (Tables S1 and S2). Because the presence of mosaic sequences due to genetic recombination would cause phylogenetic methods to produce misleading results (McGuire *et al.*, 1997), we first confirmed that significant recombination events were not detected among the 257 PPV‐D isolates using RDP4 software (Martin *et al.*, 2015). AL11pl, a PPV‐Ancestor Marcus (An) isolate, was employed as an outgroup instead of PPV‐M for the same reason, that is, because the PPV‐Marcus (M) strain was suggested as a recombinant strain between PPV‐D and PPV‐An (García *et al.*, 2014). Although a recent report has suggested that recombination events occurred in the PPV‐An isolate, it remains reasonable to use the isolate as an outgroup in our analysis because PPV‐D was not involved in the recombination (Hajizadeh *et al.*, 2019).

**Figure 2:**
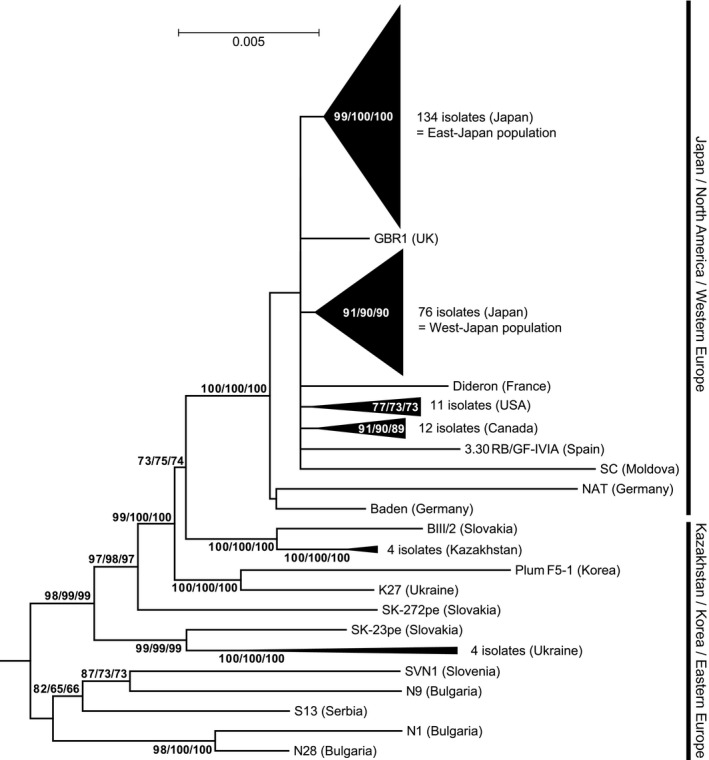
Maximum‐likelihood (ML) phylogenetic tree based on complete genome sequences of the PPV‐D strain. A PPV‐An isolate, AL11pl, was used to root the tree. For the sake of clarity, interior branches representing distinct clusters are collapsed into filled triangles. The detailed topology of the Japanese clusters is shown in Figure S1. Numbers at the nodes, or in the triangles, represent the percentage of bootstrap values obtained for 1,000 replicates in ML/neighbour‐joining/minimum‐evolution methods (values above 70% for ML are shown). The scale bar indicates the number of nucleotide substitutions per site

Because all phylogenetic trees constructed using the maximum‐likelihood (ML), neighbour‐joining (NJ), and minimum‐evolution (ME) methods showed nearly identical topologies, only the ML tree is shown in Figure 2, with bootstrap values obtained from all three methods. In these trees, a paraphyletic group consisting of isolates from Eastern Europe, Kazakhstan, and Korea was observed. The isolates from Japan, North America, and Western Europe formed a monophyletic group with an isolate from Moldova (Eastern Europe) supported by high bootstrap values (100%) in all tree‐building methods (Figure 2). In the monophyletic group, while Western European isolates branched separately, the isolates from the United States and Canada formed their own monophyletic subgroups, as described in our previous report (Maejima *et al.*, 2011). Intriguingly, Japanese isolates were not monophyletic, and formed two distinct subgroups supported by high bootstrap values in all of the trees (Figure 2); this suggested that they have distinct origins. One subgroup, which included all 37 of the previously reported Japanese isolates (Maejima *et al.*, 2011), consisted of 134 isolates mainly from the eastern region of Japan (in and around Tokyo). Conversely, the other subgroup consisted of 76 isolates mainly from western Japan (in and around Osaka), with the exception of three isolates from Adachi, Tokyo. Therefore, we referred to the former subgroup as the “East‐Japan” population, and the latter as the “West‐Japan” population. Figure S1 shows the fully expanded trees and genetic divergence of the two Japanese populations, and detailed descriptions thereof.

### 2.3 Positively selected sites in the PPV‐D genome

The phylogenetic analysis based on large‐scale genome sequencing clearly showed that there are two PPV‐D populations, East‐Japan and West‐Japan, with closely related but independent origins in Japan (Figure 2). To determine if there were distinct differences at the molecular level between these two populations, as well as between Japanese and non‐Japanese PPV‐D isolates, we evaluated the selective pressure on each codon position in the large open reading frame (ORF) and pretty interesting potyviridae ORF (PIPO) of the PPV‐D genome using the HyPhy package (Pond *et al.*, 2005) in MEGA (Tamura *et al.*, 2013). While no significant selection bias was detected in the PIPO, five amino acid residues in the large ORF appeared to be subjected to significant positive selection (Table 1). This result was supported by positive selection analyses using fixed effects likelihood (FEL) (Pond and Frost, 2005), single‐likelihood ancestor counting (SLAC) (Pond and Frost, 2005), and fast unbiased Bayesian approximation (FUBAR) (Murrell *et al.*, 2013) available on the Datamonkey server (http://www.datamonkey.org/) (Weaver *et al.*, 2018) (Table 1). Two of these positively selected residues (amino acid positions 2,303 and 2,635) were located in nuclear inclusion protein b (NIb), a potyvirus RNA‐dependent RNA polymerase. The other three residues (amino acid positions 2,855, 2,872 and 2,873) were located in the N‐terminal variable region of CP. The leucine at 2,303, cysteine at 2,635, and phenylalanine at 2,855 (aa2303L, aa2635C, and aa2855F, respectively) were conserved in the most recent common ancestor (MRCA) of the PPV‐D isolates estimated using MEGA, as well as other known PPV strains, except for the PPV‐CV strain with aa2635G (Table 1). Moreover, aa2303L was also conserved in other potyviruses, while aa2635C and aa2855F were not (Figure S2).

**Table 1:** Positive selection sites for the large ORF of the PPV‐D strain

Codon Number ^a^	Nucleotide position ^a^	Protein	*p* value and posterior probability for each method b				Amino acid residue (number of isolates)							
			HyPhy	FEL	SLAC	FUBAR	East‐Japan (134)	West‐Japan (76)	USA (11)	Canada (12)	Europe (19)	KAZ and KOR^c^ (5)	MRCA of PPV‐D	The other strains of PPV
2,303	7,053–7,055	NIb	.004	.002	.003	.989	L (130) I (3) L/I d (1)	L (69) I (3) L/I (4)	L (11)	L (12)	L (19)	L (5)	L	L
2,635	8,049–8,051	NIb	.011	.004	.014	.999	C (134)	C (10) R (56) H (8) S (2)	C (11)	C (12)	C (19)	C (5)	C	C (G in PPV‐CV)
2,855	8,709–8,711	CP	.010	.001	.011	.999	L (129) I (5)	L (75) F (1)	L (8) F (3)	L (2) F (10)	L (8) F (11)	F (5)	F	F
2,872	8,760–8,762	CP	.022	.011	.020	.971	S (131) L (2) P (1)	S (75) P (1)	S (10) A (1)	S (10) L (1) P (1)	S (18) P (1)	S (4) P (1)	S	Not conserved
2,873	8,763–8,765	CP	.042	.009	.052	.980	Q (133) R (1)	Q (73) R (2) K (1)	Q (11)	Q (11) R (1)	Q (8) R (5) P (6)	Q (5)	P	Not conserved

^a^ Position of Ou1 isolate.

^b^ Significant *p*  < .05 (HyPhy, FEL, and SLAC) and posterior probability >.95 (FUBAR) are indicated by underlining.

^c^ KAZ, Kazakhstan; KOR, Korea.

^d^ Triplet was WTA (A/T double peaks at the first base).

**Figure 3:**
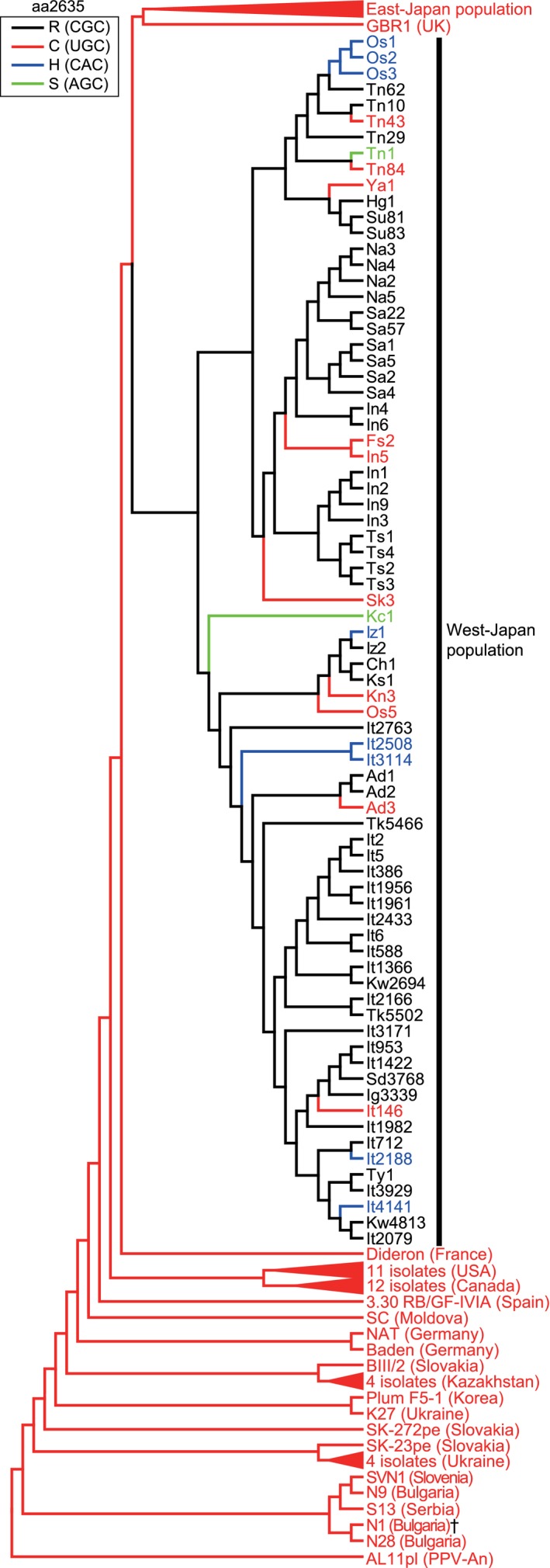
Estimated evolution of amino acid position 2,635 in the PPV‐D strain. The topology of the phylogenetic tree is the same as Figure 2, and AL11pl was used to root the tree. For the sake of clarity, interior branches representing distinct clusters sharing the same amino acid residue at position 2,635 are collapsed into filled triangles. Black, red, blue, and green branches or isolate names indicate R, C, H, and S at amino acid 2,635. ^†^N1 has UGU (synonymous substitution) as a codon of amino acid 2,635

Next, to comprehensively assess positive selection events in the PPV‐D strain, we estimated the ancestral sequences of the five positively selected residues on each node of the ML tree. Amino acid changes in NIb (at positions 2,303 and 2,635) were detected only in the Japanese isolates (Figures 3 and S3a), while those in CP (at positions 2,855, 2,872, and 2,873) were observed in both Japanese and non‐Japanese isolates (Figure S3b–d). In particular, amino acid changes at position 2,635 were detected specifically in the West‐Japan population, whereas nucleotide mutation events at the codon were not observed in the East‐Japan population or the other PPV‐D isolates, except for a synonymous mutation in the N1 isolate from Bulgaria (UGC to UGU transition) (Figure 3). Moreover, in the West‐Japan population, there were two steps involved in the amino acid change at position 2,635: first, a single nonsynonymous mutation (UGC to CGC transition) at codon 2,635, resulting in an amino acid substitution from cysteine to arginine at position 2,635 (C2635R), occurred in the MRCA of the West‐Japan population. Further nonsynonymous mutations at the codon were found in 20 of the 76 progeny isolates of the West‐Japan population. Interestingly, these mutations included 10 R2635C (CGC to UGC transition) revertants, eight R2635H (CGC to CAC transition) mutations, and two R2635S (CGC to AGC transversion) mutations (Figure 3). Strong positive selection on amino acid position 2,635 was detected within the West‐Japan population under all four tested methods (Table S3), suggesting that the amino acid changes from R to C/H/S at position 2,635 resulted from positive selection within this population.

Nonsynonymous mutations at amino acid position 2,303 were found in 10 of 210 Japanese isolates. While nucleotide substitution by transversion was far less frequent than by transition, with a transition/transversion ratio (*R*) of 5.68 in the entire genome of the PPV‐D strain, all of the amino acid substitution events at position 2,303 resulted from transversion. These events included six L2303I (UUA to AUA transversion) mutations and four L2303L/I (UUA to WUA incomplete transversion) mutations (Figure S3a). This indicates that the amino acid substitution events at position 2,303 may have conferred fitness advantages on Japanese isolates.

Amino acid changes at the three positively selected residues in the N‐terminal variable region of CP were estimated to be six L2855F, four F2855L, and one L2855I (Figure S3b); five S2872P, three S2872L, and one S2872A (Figure S3c); and four Q2873R, one P2873Q, and one Q2873K (Figure S3d).

### 2.4 Biological characterization of the positive selection sites in NIb from the Japanese PPV‐D isolates

**Figure 4:**
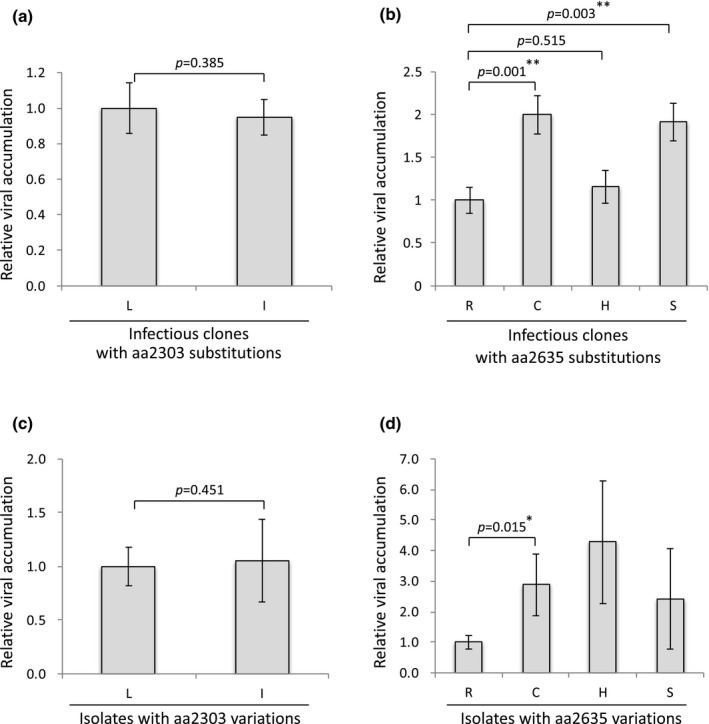
Influence of aa2303 and aa2635 on the titre of PPV. (a) and (b) Quantification of experimental PPV accumulation in *Nicotiana benthamiana*. Total RNA was extracted from leaves at 3 weeks post‐inoculation to assess systemic spread. Quantitative reverse transcription PCR (RT‐qPCR) analysis was performed on each sample. The level of endogenous 18S rRNA was used as a reference. (a) The mean level of viral RNA of the infectious clone PPV‐aa2303L was taken as a standard (1.0). The error bars represent *SEM* (*n* = 9 for both groups). Comparisons between pairs of groups were performed using the one‐tailed Welch's *t* test. (b) The mean level of viral RNA of the infectious clone PPV‐aa2635R was taken as a standard (1.0). The error bars represent *SEM* (*n* = 12 for all groups). Double asterisks indicate significantly increased viral accumulation compared to PPV‐aa2635R (one‐tailed Dunnett's test, **p < .01). (c) and (d) Quantification of natural PPV accumulation in Japanese apricot. Total RNA was extracted from leaves. RT‐qPCR analysis was performed on each sample. The level of endogenous 18S rRNA was used as a reference. (c) The mean level of viral RNA of PPV isolates with aa2303L was taken as a standard (1.0). The error bars represent *SEM* (*n* = 86 [aa2303L], and 5 [aa2303I]). Three isolates with aa2303L/I (double peak at the first base of the codon) were excluded from this analysis. Comparisons between pairs of groups were performed using the one‐tailed Welch's *t* test. (d) The mean level of viral RNA in PPV isolates with aa2635R was taken as a standard (1.0). The error bars represent *SEM* (*n* = 31 [aa2635R], 53 [aa2635C], 8 [aa2635H], and 2 [aa2635S]). PPV isolates with aa2635H and aa2635S were not included in the statistical analysis due to the relatively small sample size. Asterisk indicates significant differences compared to PPV with aa2635R (one‐tailed Welch's *t* test, *p < .05)

Because the positive selections at amino acid positions 2,303 and 2,635 were specific to the Japanese isolates among the five positively selected sites, we hypothesized that they are responsible for intra‐strain biological variations among these isolates through effects on the function of the corresponding protein NIb. Given that NIb is a potyvirus RNA‐dependent RNA polymerase (Hong and Hunt, 1996), we investigated whether amino acid substitutions at these residues under positive selection would affect viral titre in host plants. First, we constructed PPV variants at amino acid positions 2,303 (PPV‐aa2303L and PPV‐aa2303I) and 2,635 (PPV‐aa2635C, PPV‐aa2635R, PPV‐aa2635H, and PPV‐aa2635S), and measured viral titre by quantitative reverse transcriptionPCR (RT‐qPCR) in *Nicotiana* *benthamiana*, an experimental host of PPV. At 3 weeks post‐inoculation (wpi), PPV‐aa2303I did not outperform PPV‐aa2303L in terms of viral accumulation at the systemic level (Figure 4a). In contrast, PPV‐aa2635C and PPV‐aa2635S were accumulated at significantly higher levels than PPV‐aa2635R systemically, while PPV‐aa2635H was not (Figure 4b). We confirmed that the systemically infecting viruses retained the same point mutations as the corresponding inocula by RT‐PCR and direct sequencing. These results suggested that the amino acid residue at position 2,635 affects the level of PPV accumulation.

The amino acid position 2,635‐dependent difference in systemic viral accumulation raised the question of whether the amino acid residue at this positively selected site also affected viral competitiveness. To evaluate the relative competitiveness of the amino acid position 2,635 variants in terms of systemic spread, we performed a competition assay by simultaneous inoculation with PPV‐aa2635R and each of PPV‐aa2635C, PPV‐aa2635H, or PPV‐aa2635S. At 3 wpi, 75% (9/12) of the plants inoculated with PPV‐aa2635R and PPV‐aa2635H retained mixed infection, while 17% (2/12) and 8% (1/12) were dominantly infected by PPV‐aa2635R and PPV‐aa2635H, respectively, in leaves infected systemically (Table 2). In contrast, among the plants inoculated with PPV‐aa2635R and PPV‐aa2635C, 67% (8/12) were dominantly infected by PPV‐aa2635C, and the rest retained mixed infections; no plant was dominantly infected by PPV‐aa2635R. We obtained similar results from the plants inoculated with PPV‐aa2635R and PPV‐aa2635S (58% were dominantly infected by PPV‐aa2635S and the rest retained mixed infection). These results suggest that the amino acid substitutions from arginine to cysteine or serine at position 2,635 contribute to higher levels of viral competitiveness at the systemic level. Although the same assay was performed on amino acid position 2,303 using *N. benthamiana*, PPV‐aa2303I was not superior to PPV‐aa2303L in terms of dominance at the systemic level (Table S4).

**Table 2:** Competitive assay of mix‐inoculated PPV aa2635 variants

Inoculated PPV	*n*	Major sequence of aa2635 in upper leaves at 3 weeks post‐inoculation (number of plants)
aa2635C + aa2635R	12	C (8), C + R (4)
aa2635H + aa2635R	12	H (2), H + R (9), R (1)
aa2635S + aa2635R	12	S (7), S + R (5)
aa2635C	12	C (12)
aa2635R	12	R (12)
aa2635H	12	H (12)
aa2635S	12	S (12)

Finally, to further evaluate the role of amino acid position 2,635 under natural conditions, we quantified viral titre in the original host plants by RT‐qPCR, using total RNA from field‐collected Japanese apricot plants infected with 48 isolates of the East‐Japan population and 46 isolates of the West‐Japan population. Although no correlation was detected between amino acid position 2,303 and viral titre (Figure 4c), intriguingly, the viral titre was significantly higher in hosts infected with aa2635C‐type PPV than in those infected with aa2635R‐type PPV (Figure 4d). These results suggested that amino acid substitutions at position 2,635 associated with positive selection predominantly controls the accumulation of Japanese PPV isolates in the natural host.

## 3 DISCUSSION

A number of studies on the molecular epidemiology of PPV have aimed to identify viral genomic signatures explaining biological features of the virus (Bousalem *et al.*, 1994; Dallot *et al*, 2001; Glasa *et al.*, 2002, 2004, 2013; Maejima *et al.*, 2011; Schneider *et al.*, 2011; Chirkov *et al.*, 2018). However, identification of such genomic determinants at the single amino acid level has been unsuccessful owing to the high sequence divergence among strains (Salvador *et al.*, 2008), even among populations or isolates within a single strain (except for certain obvious point mutations associated with aphid transmissibility) (Maiss *et al.*, 1995; Kamenova *et al.*, 2002; Glasa *et al.*, 2004). In the current study, we demonstrate for the first time that the specific amino acid residue under positive selection determines intra‐strain biological variation in PPV. Our results indicate that genome sequence information enables not only conventional strain typing and phylogenetic/phylogeographic analysis, but also biological characterization of plant viruses at the population and isolate levels. In particular, the combination of phylogeny and amino acid substitution events at positively selected sites helps to estimate how viral amino acid changes contributed to the dissemination of the virus population, providing deeper insight into the molecular epidemiology of plant viruses.

### 3.1 Origin and dispersal routes of PPV‐D in Japan

This study revealed that all 210 Japanese isolates, which were collected throughout most of the areas where PPV was found from 2008 to 2014, belong to the PPV‐D strain, but are divided into two genetically distinct populations (East‐Japan and West‐Japan) (Figure 2). Both Japanese populations were relatively close to Western European PPV‐D, suggesting that they originated in Western Europe. In contrast, the Korean isolate was closely related to Eastern European isolates (Figure 2). This suggests that PPV‐D in Korea (Oh *et al.*, 2017) has a different origin from the PPV‐D populations in Japan, even though these two Asian countries are geographically close.

In the East‐Japan population, the Tokyo isolates sampled in and around Ome city were distributed in all six subclades with the highest sequence diversity (Figure S1a, c), suggesting that this area was the source of the plum pox disease outbreak in the East‐Japan region. Supporting this theory, there are trading records of scions or nursery stocks from Ome city to remote cities such as Odawara, Mito, Nagahama, Suita, and Nara, where isolates belonging to the East‐Japan population were identified. In the West‐Japan population, only isolates from Osaka prefecture were distributed in all three clades and the ungrouped branch (Figure S1b); these isolates had the greatest sequence diversity (Figure S1c). However, the origins and dispersal routes of the West‐Japan population remain unknown. Further sample collection and sequence analysis may answer this question.

### 3.2 Evidence of intra‐strain positive selection in PPV

Our study provides strong evidence of intra‐strain positive selection in PPV. Thus far, there is minimal evidence and there have been no prospective studies of positive selection acting on PPV; however, Glasa *et al.* reported weak positive selection at two codon sites in the C‐terminal region of P3 protein based on comparison of three PPV strains (Glasa *et al.*, 2002), and Hajizadeh *et al*. recently reported four positively selected codon sites in the PIPO of the PPV‐D strain (Hajizadeh *et al.*, 2019). In this study, comparative genomic analysis of 257 PPV‐D isolates detected significant positive selection, in at least three of the four tested methods, at five codon sites: three (amino acid positions 2,855, 2,872, and 2,873) resided in the N‐terminal region of CP, and two (amino acid positions 2,303 and 2,635) resided in NIb.

The N‐terminal region of CP, which is extremely variable among different potyviruses (Figure S2), is involved in long‐distance viral movement and aphid transmission (Salvador *et al.*, 2006). Several sites within this region are related to the infectivity of PPV to *Nicotiana* plants (Carbonell *et al.*, 2013). However, these functional sites were not identical to the three positively selected sites detected in this study. Because amino acid substitutions driven by positive selection in the N‐terminal region of CP were observed in geographically dispersed PPV‐D isolates (Figure S3b–d), elucidation of the biological roles of these positively selected sites is expected to shed light on the global evolution of the PPV‐D strain. It is noteworthy that the three positively selected sites were located within the epitope region (amino acid region 2,852–2,874) recognized by the PPV‐D strain‐specific monoclonal antibodies 4DG5 and 4DG11 (Candresse *et al.*, 2011). Therefore, it would be valuable to determine whether amino acid substitutions at these sites affect the antigenicity of the virus and compromise diagnosis and strain typing by the monoclonal antibodies.

The amino acid substitution from leucine to isoleucine at position 2,303 (UUA to AUA transversion) in NIb occurred independently several times in both the East‐Japan and West‐Japan populations (Table 1 and Figure S3a). This substitution was not detected for non‐Japanese PPV‐D isolates, nor in any other PPV strain (Table 1). This indicates that the positive selection at amino acid position 2,303 has important implications for the evolution of PPV in Japan. However, PPV‐aa2303I did not outperform PPV‐aa2303L in terms of systemic accumulation (Figure 4a), or in the competitive assay (Table S4) using the experimental host *N. benthamiana*. Because the only discernible difference between the Japanese and non‐Japanese PPV‐D isolates is whether or not the major host plant was Japanese apricot (Tables S1 and S2), the L2303I amino acid substitution may be involved in the adaptation of PPV to Japanese apricot. Although the systemic accumulation of PPV with aa2303I was not greater than that of PPV with aa2303L in Japanese apricot, it should be noted that this result was based on rather limited numerical data (only five isolates with aa2303I compared to 86 isolates with aa2303L) (Figure 4c). Inoculation tests using a larger sample size may clarify the significance of the positive selection at amino acid position 2,303 for the evolution of the PPV‐D strain.

### 3.3 Biological and epidemiological implications of amino acid position 2,635

The positive selection at amino acid position 2,635 in NIb was peculiar to the West‐Japan population (Table 1), where the amino acid substitution events were divided into two stages. The first amino acid substitution (C2635R) occurred in the MRCA of the West‐Japan population, and the second substitution (R2635C/H/S) occurred frequently in its progeny (Figure 3). Interestingly, the viral accumulation level for the isolates with aa2635C was significantly higher than for aa2635R‐type isolates in Japanese apricot, the natural host (Figure 4d). This is consistent with the results of assays using the experimental host *N. benthamiana* (Figure 4b). Because amino acid position 2,635 is in the vicinity of a conserved Gly‐Asp‐Asp (GDD) motif that is essential for the activity of NIb (Li and Carrington, 1995), amino acid position 2,635 may contribute to the efficiency of virus replication.

It has been demonstrated that there is a significant positive correlation between viral accumulation level and the efficiency of insect acquisition/transmission (Simons, 1958; Pirone and Megahed, 1966; Banik and Zitter, 1990; Escriu *et al.*, 2000; Matsukura *et al.*, 2013; Li *et al.*, 2014) including potyviruses (De Bokx *et al.*, 1978; Romanow *et al.*, 1986; Wosula *et al*., 2012). This has led us to postulate that aa2635C‐type PPV is more readily transmitted by aphids than aa2635R‐type PPV. Furthermore, in the competitive assay of the amino acid position 2,635 variants, PPV‐aa2635C was more abundant in systemically infected leaves than PPV‐aa2635R (Table 2), suggesting that aa2635C‐type PPV is more likely to be acquired and transmitted by aphid vectors than aa2635R‐type PPV, even in coinfection conditions. aa2635C‐type PPV is therefore considered to be more infectious than aa2635R‐type PPV, given that its high titre and competitiveness in plants is probably associated with high aphid transmissibility. Another variant, PPV‐aa2635S, was also significantly superior to PPV‐aa2635R in terms of viral titre (Figure 4b) and competitiveness (Table 2) in *N. benthamiana*, implying that the aa2635S‐type PPV also has higher epidemic potential than aa2635R‐type PPV. Although there was no significant difference in accumulation levels between PPV‐aa2635H and PPV‐aa2635R in *N. benthamiana*, the isolates with aa2635H tended to accumulate at higher levels than those with aa2635R in the natural host, Japanese apricot (Figure 4d).

Considering the biological features associated with amino acid position 2,635, and its evolutionary history in the PPV‐D strain, although the MRCA of the West‐Japan population harbouring aa2635R has been less epidemic than the East‐Japan population (aa2635C‐type), several progeny of the former population are becoming as epidemic as the East‐Japan population due to independent acquisition of an amino acid substitution at position 2,635 (R2635C, S, or possibly H). This suggests that genotyping of amino acid position 2,635 is a useful tool for evaluating the individual epidemiological features of PPV‐D in Japan.

### 3.4 Possible contribution of reversible evolution to geographical dissemination

The significant positive selection at amino acid position 2,635 in the West‐Japan population (Table S3), as well as experimental evidence from reverse genetics (Figure 4b and Table 2) and field samples (Figure 4d), strongly suggest that aa2635R‐type PPV is less adaptive than aa2635C‐type PPV. This raises the following question: why did the MRCA of the West‐Japan population acquire the reversible amino acid change from cysteine to arginine?

Non‐adaptive amino acid changes followed by back mutation (reversion) at positively selected sites are known as “reversible evolution”, and have been reported for epitopes of several human viruses (Leslie *et al.*, 2004; Timm *et al.*, 2004; Botosso *et al.*, 2009). In such cases, the less adaptive amino acid change contributes to viral escape from host immune responses by altering viral antigenicity, and reversion to restore the original antigens occurs after infection of new hosts that have not yet developed immunity to them. This is because viruses with immune escape mutations are inherently less adaptive than those with the original antigens in the absence of immune pressure.

For plant viruses, including PPV, international and domestic pest control by plant quarantine regulations is a significant barrier to their dissemination to new countries and areas. Among PPV strains, only PPV‐D seems to be a quarantine‐escaped strain that has successfully spread worldwide, including to Japan. Schneider *et al.* speculated that this may be due to the latent nature of PPV‐D infections; specifically, relatively low titres and reduced transient symptoms make PPV‐D‐infected trees difficult to identify and eradicate, although intra‐strain biological variation in symptom severity is present in the PPV‐D strain (Schneider *et al.*, 2011). Because the accumulation level of PPV in *Prunus* leaves also correlates with symptoms (Martínez‐Gómez and Dicenta, 2001; Salvador *et al.*, 2008; Maejima *et al.*, 2014), our study suggests that aa2635R‐type PPV‐D causes further attenuated symptoms compared with canonical PPV‐D, including the East‐Japan population, which harbours aa2635C. Therefore, we formulated the following hypothesis for the emergence of reversible evolution at amino acid position 2,635 in the West‐Japan population: the less adaptive amino acid change C2635R contributed to the escape of the MRCA of the West‐Japan population from quarantine by transiently enhanced attenuation; the adaptive reversion R2635C (to restore the original level of epidemicity) occurred in its progeny after introduction into Japan. Genetic drift with the so‐called founder effect could also have contributed to the introduction of the less‐adaptive MRCA of the West‐Japan population into Japan. To the best of our knowledge, this study provides the first molecular evidence of reversible evolution of plant pathogens that may be related to a mechanism for overcoming plant quarantine regulations. A recent study reported that the incubation period (time from infection to symptom expression) and latent period (time from infection to onset of infectiousness) are almost synchronous for PPV (Rimbaud *et al.*, 2015a). It will be interesting to see how the amino acid substitutions at position 2,635 affect these features.

## 4 EXPERIMENTAL PROCEDURES

### 4.1 Sample collection

From May 2009 to August 2014, 210 samples of *Prunus* leaves, petals, and branches infected with PPV, including 37 samples reported in our previous study (Maejima *et al.*, 2011), were collected from most of the cities/towns in Japan where PPV has been detected (Figure 1). Collection was carried out with permission from the Ministry of Agriculture, Forestry and Fisheries (MAFF) of Japan. Viral infection was confirmed by MAFF Plant Protection Stations (PPS) using both the immunochromatography assay kit (Nippon Gene) and the RT‐LAMP assay kit (Nippon Gene). Table S1 shows the data for the Japanese samples (isolate name, geographical origin, host plant, year of sampling, and GenBank accession number). A PPV‐D‐infected sample from the UK (GBR1) was kindly provided by MAFF PPS. Other PPV‐D‐infected European samples, from Bulgaria, Serbia, and Slovenia (N1, N9, N28, S13, and SVN1), were described previously (Maejima *et al.*, 2014). These non‐Japanese samples were imported with permission from MAFF. All samples were stored at −80 °C until use.

### 4.2 Sequencing of the viral genome

Genome sequences of PPV isolates were determined as described previously (Maejima *et al.*, 2011). The 24 nt at the 5′ end and 26 nt at the 3′ end corresponding to the primer sequences used for RT‐PCR amplification were not determined. Mixed infection was improbable because very few ambiguous sites (average 0.67 sites per isolate), and no discrepancy in the overlapping region of the amplified fragments, were identified among the 173 Japanese isolates and six non‐Japanese isolates sequenced in this study.

### 4.3 Phylogenetic analysis

The 210 genome sequences of the Japanese isolates were aligned with those of 47 non‐Japanese PPV‐D isolates and a PPV‐An isolate, as an outgroup sequence (Table S2), using MEGA 6 (Tamura *et al.*, 2013). Recombination among sequences was analysed using the RDP (Martin and Rybicki, 2000), GENECONV (Padidam *et al.*, 1999), BOOTSCAN (Salminen *et al.*, 1995), MAXCHI (Smith, 1992), CHIMAERA (Posada and Crandall, 2001), SISCAN (Gibbs *et al.*, 2000), and 3SEQ (Boni *et al.*, 2007) methods in RDP4 (Martin *et al.*, 2015), with the default settings and a *p* = .05 threshold. Events detected by fewer than two methods were ignored. The best nucleotide substitution model (GTR + G+I) was selected using MEGA 6. Phylogenetic analysis was performed with MEGA 6 using the ML method under the GTR model, with the complete deletion option for gap sites. The robustness of the tree topology was confirmed using NJ and ME algorithms with MEGA 6. The ancestral sequences of PPV‐D isolates were estimated using the phylogenetic tree constructed using the ML method. The phylogenetic trees were edited using Adobe Illustrator CS4.

### 4.4 Selection, transition/transversion ratio, and genetic divergence analysis

Natural selection was estimated for each codon of the large ORF (nt 147–9,569) and PIPO (nt 2,915–3,217) of 257 PPV‐D genome sequences using the Hyphy package under the General Time Reversible model (Nei and Kumar, 2000) in MEGA 6, and using FEL, SLAC, and FUBAR methods on the Datamonkey server. The transition/transversion ratio (*R*) was estimated for the aligned PPV‐D complete genome sequences using the ML method with MEGA 6. For the analysis of genetic divergence within each geographical group of Japanese populations, the number of base substitutions per site was calculated using a pairwise distance matrix in MEGA 6, and was plotted on a graph using Microsoft Excel.

### 4.5 Site‐directed mutagenesis

Point mutations were introduced into amino acid residues 2,303 and 2,635 of an agroinfectious clone, pPPVOu (Maejima *et al.*, 2014), using the GeneArt Site‐Directed Mutagenesis System (Thermo Fisher Scientific). The original pPPVOu is referred to as PPV‐aa2303L and PPV‐aa2635C in the present study, because it was constructed based on a PPV‐D isolate from Ome city harbouring aa2303L and aa2635C. The primer pairs used to introduce mutations were as follows: CGC‐F (5′‐CATCCGGATACACACGATcGCATTTGTCGGTACTTCGTGA‐3′) and CGC‐R (5′‐TCACGAAGTACCGACAAATGCgATCGTGTGTATCCGGATG‐3′) for PPV‐aa2635R; CAC‐F (5′‐CATCCGGATACACACGATcaCATTTGTCGGTACTTCGTGA‐3′) and CAC‐R (5′‐TCACGAAGTACCGACAAATGtgATCGTGTGTATCCGGATG‐3′) for PPV‐aa2635H; AGC‐F (5′‐CATCCGGATACACACGATaGCATTTGTCGGTACTTCGTGA‐3′) and AGC‐R (5′‐TCACGAAGTACCGACAAATGCtATCGTGTGTATCCGGATG‐3′) for PPV‐aa2635S; and ATA‐F (5′‐CTCAGAGATAGAaTAGAAGGAAA‐3′) and ATA‐R (5′‐TTTCCTTCTAtTCTATCTCTGAG‐3′) for PPV‐aa2303I (mutated sites are shown in lowercase letters). The sequences of the mutants (PPV‐aa2303I, PPV‐aa2635R, PPV‐aa2635H, and PPV‐2635S) were checked as described above.

### 4.6 Inoculation and detection

*Agrobacterium tumefaciens* carrying the plasmid pPPVOu (PPV‐aa2635C/PPV‐aa2303L) or its mutants (PPV‐aa2303I, PPV‐aa2635R, PPV‐aa2635H, and PPV‐2635S) was harvested and resuspended in infiltration buffer (Takahashi *et al.*, 2006) to an optical density at 600 nm of 1.0. Each suspension was then diluted 1,000‐fold with the same buffer to mimic natural infection with a small number of infection sites. For viral accumulation assays, *N. benthamiana* was independently agroinfiltrated with each suspension. For virus competition assays, *N. benthamiana* was agroinfiltrated with 1:1 mixed suspensions (PPV‐aa2303L and PPV‐aa2303I; PPV‐aa2635R and PPV‐aa2635C; PPV‐aa2635R and PPV‐aa2635H; or PPV‐aa2635R and PPV‐aa2635S). At 3 wpi, total RNA was purified from the seventh to ninth leaves above the inoculated leaf using ISOGEN (Nippon Gene). For viral accumulation assays, RT‐qPCR was conducted as described below. For both assays, the viral sequences at amino acid positions 2,303 and 2,635 were checked by RT‐PCR with primers PPV6600F (5′‐CAAATTTCCAAACGAAGAGC‐3′) and PPV9039R (5′‐CGCTTAACTCCTTCATACCAAGT‐3′), followed by direct sequencing with primers PPV7383R (5′‐GCTGCTTTCATGTTAAGTGC‐3′) and PPV7684F (Maejima *et al.*, 2011), respectively, as previously described (Maejima *et al.*, 2014). In Sanger sequencing, when the peak height of a nucleotide was at least 2‐fold greater than those of the other nucleotides at each base, it was considered to be dominant.

### 4.7 Quantification of viral genome RNA

RT‐qPCR analysis was performed as described previously (Komatsu *et al.*, 2010). The primer sets used for RT‐qPCR were PPVR7F and PPVR7R (Maejima *et al.*, 2014) for PPV RNA, PR1F and PR2R (Feng *et al.*, 2006) for 18S rRNA of *P. mume*, and Nb18S‐193F and Nb18S‐280R (Hashimoto *et al.*, 2012) for 18S rRNA of *N. benthamiana*.

## Supporting information


**FIGURE S1** Fully expanded ML trees and genetic divergence of the East‐Japan and West‐Japan populations. Numbers at the nodes represent the percentage of bootstrap values obtained for 1,000 replicates using the ML method (only values above 70% are shown). The scale bar indicates the number of nucleotide substitutions per site. (a) Tree of the East‐Japan population. The East‐Japan population consisted of two clades (A and B), as reported in our previous study (Maejima *et al*., 2011). In this population, the isolates sampled in and around Ome city, where PPV was first found in Japan, belonged to every subclade and included all of the ungrouped isolates. Clade A was further divided into four subclades (A‐I, A‐II, A‐III, and A‐IV) with high bootstrap values and four ungrouped isolates (Ak1, Ok1, Ou6, and Ou16). Subclade A‐I consisted of 46 isolates from 6 out of 11 prefectures where PPV was found: Kanagawa (Od), Ibaraki (Mi), Nara (Na), Osaka (Su), Shiga (Ng), and Tokyo (Ou). Interestingly, all of the isolates in subclade A‐I were found in ornamental Japanese apricot gardens and all of the Tokyo isolates in the subclade were collected from an ornamental Japanese apricot garden (OuX) in Ome city. As shown in our previous study (Maejima *et al*., 2011), viruses from the same cultivars (cvs.) were closely related to each other: cvs. Kurenai (Od2 and OuX2‐1 [Ou14]), Tenjinbai/Kusudama (Od1, OuX12‐1, and OuX10‐1), Hamachidori (Mi1, Mi2, Mi3, and OuX4‐3 [Ou15]), and Mongakushi (Od4, and OuX3‐6). This indicated that the infected scions in Kanagawa (Od) and Ibaraki (Mi) were derived from mother trees in the ornamental garden (OuX) in Ome city. MAFF’s investigation of trade records revealed that these infected Japanese apricot trees in Nara (Na), Osaka (Su), and Shiga (Ng) were also introduced from Ome city (Ou), indicating that the infected plant materials from Ome city were the source of infection in these western Japanese regions. Subclade A‐II consisted of four isolates from Tokyo (Ak and Ou) and an isolate from Saitama (Hn). Hn2, Ou17, and Ou18, which clustered with high bootstrap values, were found in a boundary area between Tokyo and Saitama. Subclade A‐III consisted of 50 isolates: 35 isolates from Tokyo (Ak, Hi, Hk, Ko, Ok, and Ou), three from Saitama (Hn and Ni), seven from Ibaraki (Kg), and five from Wakayama (Wa). The seven Kg isolates (Koga city, Ibaraki), five Wa isolates (Wakayama city, Wakayama), and eight Hk isolates (Higashikurume city, Tokyo) formed their own monophyletic clusters, indicating that single viral introduction events resulted in the occurrence of PPV in these areas. Ni1 and Ni2, which were found in Niiza city (southeastern area of Saitama), were closely related to Ou29. Ou29 and Ou28 are found in Japanese apricot trees grown by a supplier of nursery stock in Ome city, suggesting that PPV found in Niiza city is derived from the nursery stock(s) of Ome city. Subclade A‐IV is a newly recognized group consisting of four isolates from the ornamental garden (OuX) in Ome city, and an isolate found in the same neighbourhood. Clade B was further divided into two subclades (subclades B‐I and B‐II) with high bootstrap values, and two ungrouped isolates (Ak3 and Hi2); this was consistent with our previous study (Maejima *et al*., 2011). Subclade B‐I consisted of 15 isolates from Tokyo (Ak, As, Fu, Ha, Hi, and Ou) and an isolate from Ibaraki (Mi). Mi5 clustered with two isolates from the ornamental garden (OuX) in Ome city. Three isolates from Akishima city formed their own monophyletic cluster. Subclade B‐II consisted of five isolates from Tokyo (Ha and Ou) and an isolate from Saitama (Ir). Ir1 and Ou20, which clustered with high bootstrap values, were found in a boundary area between Tokyo and Saitama. (b) Tree of the West‐Japan population. The West‐Japan population consisted of three clades (clades X, Y, and Z) with high bootstrap values, and an ungrouped isolate (Kc1). In this population, the isolates from Osaka exclusively constituted clade X, branched at the roots of clades Y and Z, and included all of the ungrouped isolates. Clade X consisted entirely of isolates from Osaka (Hg, Os, Su, Tn, and Ya) and was further divided into two subclades (subclades X‐I and X‐II). Subclade X‐I consisted of isolates from two adjacent cities, Osakasayama (Os) and Tondabayashi (Tn), located in the southeastern part of Osaka. Three isolates from Osakasayama city (Os1, Os2, and Os3) formed a monophyletic cluster. Subclade X‐II consisted of two isolates (Su81 and Su83) from private gardens in Suita city located near the ornamental garden where Su1, 3, 14, 46, and 49 (subclade A‐I) were collected, as well as single isolates from Yao city and Higashiosaka city (Ya1 and Hg1, respectively). Clade Y was composed of three subclades (subclades Y‐I, Y‐II, and Y‐III) and an ungrouped isolate (Sk3). Subclade Y‐I consisted of 10 isolates from Nara (Na and Sa) forming a monophyletic cluster and four isolates from Aichi (Fs and In). The other isolates from Aichi (In) formed a monophyletic cluster, subclade Y‐II. Subclade Y‐III consisted of four isolates from Mie (Ts). Clade Z was composed of two subclades (Z‐I and Z‐II) and an ungrouped isolate (Os5). Subclade Z‐I consisted of five isolates from Osaka (CH, Iz, Kn, and Ks). Subclade Z‐II consisted of three isolates from Tokyo (Ad), an isolate from Osaka (Ty), and 29 isolates from Hyogo (Ig, It, Kw, Sd, and Tk; collected from 26 different Japanese apricot nurseries managed by 16 farmers to avoid sampling bias). It2763 first branched out of the subclade Z‐II, and three Ad isolates (Adachi ward, Tokyo) and Ty1 isolate (Tondabayashi city, Osaka) were included in a monophyletic subcluster formed by the other isolates from Hyogo; this indicated that the PPV found in Adachi ward and Tondabayashi city were derived from nursery stock(s) introduced from Hyogo. (c) Genetic divergence within each geographical group of the East‐ and West‐Japan populations. The pairwise comparison matrices of genetic divergence (number of base substitutions per site) within each geographical group were plotted. The maximum genetic divergence for each geographical group is shown on the right side of the graph. Numbers in parentheses are the numbers of virus isolates comprising each geographical group

**FIGURE S2** Amino acid alignments of the large open reading frame of PPV and other potyviruses at positions corresponding to the five positively selected residues detected from the PPV‐D strain. The background indicates the percentage of amino acid similarity: black, 100%; dark grey, 80%; light grey, 60%

**FIGURE S3** Estimated evolution of aa2303 (a), aa2855 (b), aa2872 (c), and aa2873 (d) of the PPV‐D strain. The topology of phylogenetic tree is the same as Figure 2, and AL11pl was used to root the tree. The colours of branches and isolate names indicate the amino acid residues of the ancestors and isolates, respectively. ^†^L (UUG); ^††^ S (UCU); ^†††^P (CCA)

**TABLE S1** List of Japanese PPV‐D isolates used in this study

**TABLE S2** List of non‐Japanese PPV isolates used in this study

**TABLE S3** Positive selection sites for the large open reading frame of the West‐Japan population

**TABLE S4** Competitive assay of mix‐inoculated PPV aa2303 variants

## Data Availability

The data supporting these findings are available in DDBJ/EMBL/GenBank at https://www.ncbi.nlm.nih.gov/genbank/ under the accession numbers LC374954–LC375132.
