# Supplementary figures and images for: Intra‐strain biological and epidemiological characterization of plum pox virus
Source: Mol Plant Pathol. 2020 Jan 24;21(4):475–88. doi: 10.1111/mpp.12908 (PMC7060144; doi:10.1111/mpp.12908)

Fig. S1 Maejima *et al.*

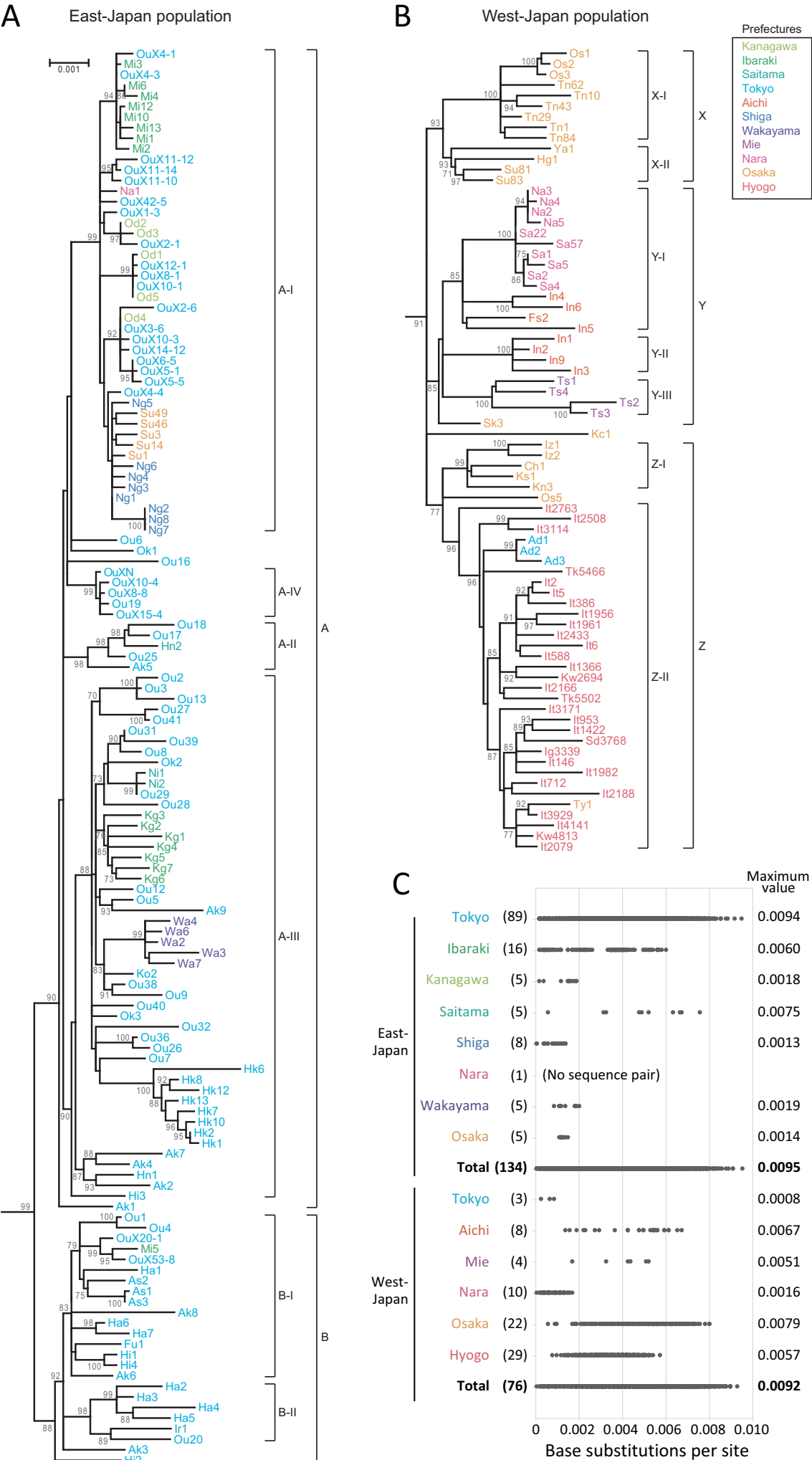

Supplement: Supplementary file 2 [file MPP-21-475-s002.pdf]

Fig. S2 Maejima *et al.*

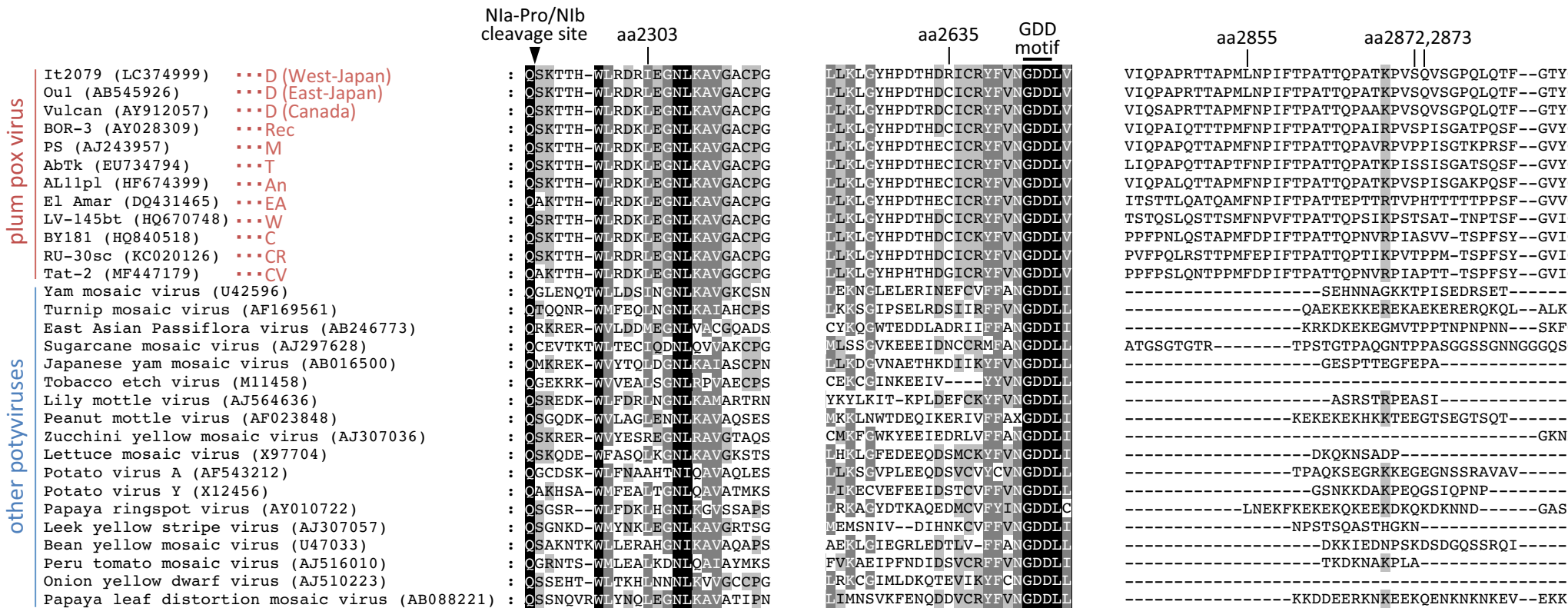

Supplement: Supplementary file 4 [file MPP-21-475-s004.pdf]

Fig. S3 Maejima *et al.*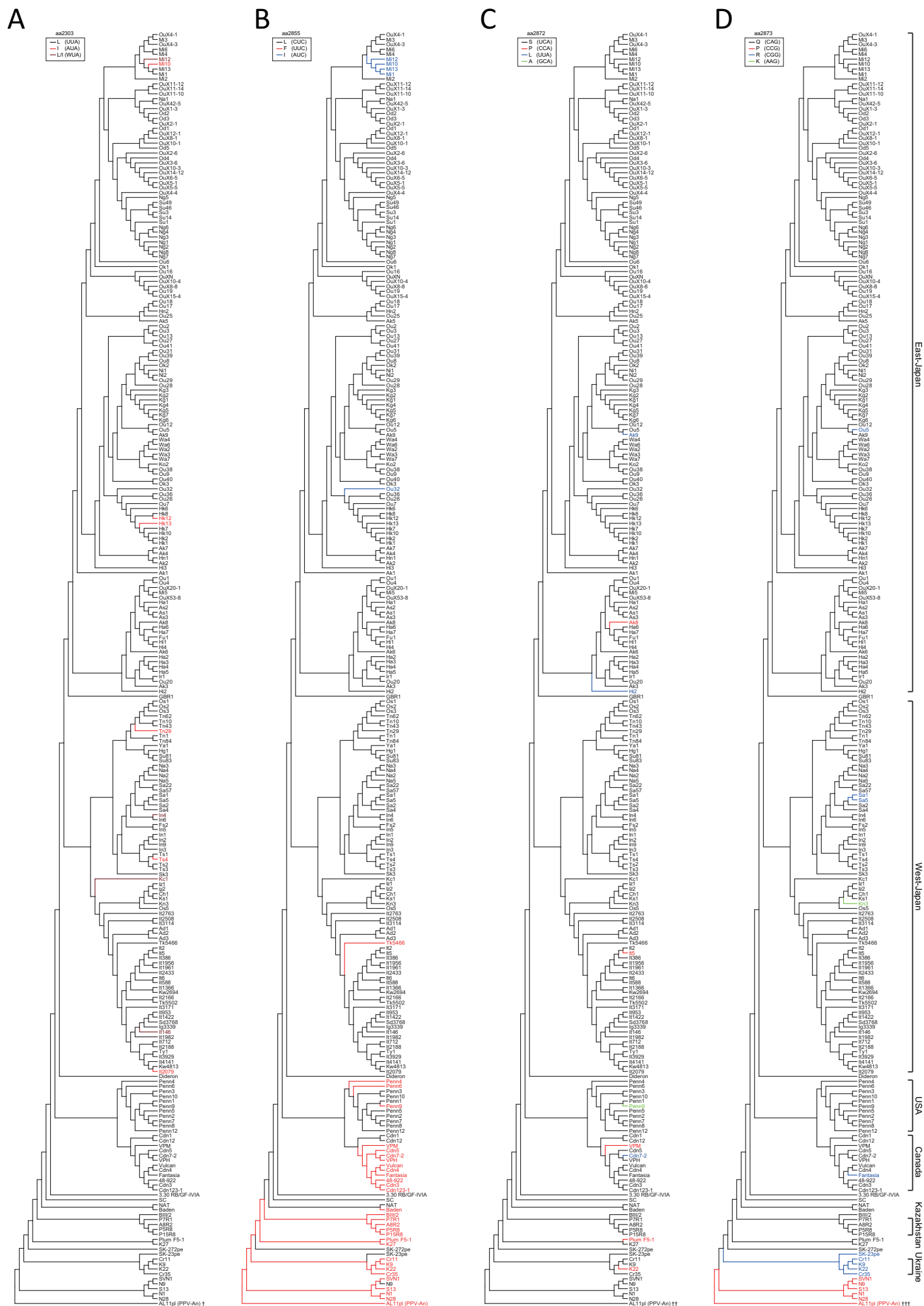

Supplement: Supplementary file 6 [file MPP-21-475-s006.pdf]
